# Context-Dependent Gestural Laterality: A Multifactorial Analysis in Captive Red-Capped Mangabeys

**DOI:** 10.3390/ani12020186

**Published:** 2022-01-13

**Authors:** Juliette Aychet, Noémie Monchy, Catherine Blois-Heulin, Alban Lemasson

**Affiliations:** 1 Univ Rennes, Normandie Univ, CNRS, EthoS (Éthologie Animale et Humaine)—UMR 6552, 35380 Paimpont, France; noemie.monchy@etudiant.univ-rennes1.fr (N.M.); catherine.blois-heulin@univ-rennes1.fr (C.B.-H.); alban.lemasson@univ-rennes1.fr (A.L.); 2 Institut Universitaire de France, CEDEX 05, 75231 Paris, France

**Keywords:** gestures, catarrhine monkeys, manual laterality, social laterality, emotional lateralization, language origins

## Abstract

**Simple Summary:**

In vertebrates, each of the two hemispheres of the brain controls the opposite side of the body. Consequently, lateralized actions in animals may reflect that one hemisphere processes particular functions underlying the behavior. In humans, for instance, the left-brain hemisphere is specialized for language functions. In several species of non-human primates, communicative gestures are preferentially produced with the right hand. It has been hypothesized that the human language lateralization has common evolutionary origins with a left-hemispheric specialization for gestures in African-Asian primates. Therefore, it is particularly interesting to describe the gestural laterality of non-human primates to understand the evolutionary history of intentional communication. Previous studies reported different factors affecting the gestural laterality of great apes, but this has rarely been investigated in more distant species of monkeys. In the present study, we observed the manual gestures produced by captive mangabeys. We found that, although monkeys were not lateralized when considering their gestures, on the whole, they preferentially gesture with their right hand in contexts of aggression or submission, and that the position of their receiver also affected the hand used. As for chimpanzees, gorillas, or humans, the gestural laterality of mangabeys depends on the context in which they communicate.

**Abstract:**

Catarrhine primates gesture preferentially with their right hands, which led to the hypothesis of a gestural origin of human left-hemispheric specialization for language. However, the factors influencing this gestural laterality remain understudied in non-hominoid species, particularly in intraspecific contexts, although it may bring valuable insights into the proximate and ultimate causes of language lateralization. We present here a preliminary investigation of intraspecific gestural laterality in catarrhine monkeys, red-capped mangabeys (*Cercocebus torquatus*). We described the spontaneous production of brachio-manual intentional gestures in twenty-five captive subjects. Although we did not evidence any significant gestural lateralization neither at the individual- nor population-level, we found that mangabeys preferentially use their right hands to gesture in negative social contexts, such as aggressions, suggesting an effect of emotional lateralization, and that they adapt to the position of their receiver by preferentially using their ipsilateral hand to communicate. These results corroborate previous findings from ape studies. By contrast, factors related to gesture form and socio-demographic characteristics of signaler and receiver did not affect gestural laterality. To understand better the relationships between gestural laterality and brain lateralization from an evolutionary perspective, we suggest that the gestural communication of other monkey species should be examined with a multifactorial approach.

## 1. Introduction

Humans exhibit a left-hemispheric specialization for language functions, particularly through Broca’s and Wernicke’s brain areas, which results in a right-biased lateralization for verbal and non-verbal communicative behaviors [1,2,3,4,5,6,7]. Great apes also exhibit neuroanatomical asymmetries in favor of the left hemisphere in brain regions homologous to Broca’s area (in chimpanzees, *Pan troglodytes*, bonobos, *Pan paniscus*, and gorillas, *Gorilla gorilla* [8]), and functional association of these regions with gestural communication have been evidenced in chimpanzees [9,10,11]. A communicative gesture can be defined as a non-locomotor and mechanically ineffective brachio-manual signal, that is directed to a recipient and lead to a voluntary response from the latter [12,13,14,15,16,17,18,19,20,21], although it has to be noted that this operational definition may vary depending on the authors [22]. Population-level right-handedness for the production of communicative gestures has been evidenced in several catarrhine primates (e.g., chimpanzees [23,24,25,26], gorillas [27,28,29], Tonkean macaques, *Macaca tonkeana* [30,31], olive baboons, *Papio anubis* [31,32,33]). Moreover, gestural laterality is dissociated from hand preference observed in non-communicative actions in numerous species (e.g., in chimpanzees [26], Campbell’s mona monkeys, *Cercopithecus campbelli*, and red-capped mangabeys, *Cercocebus torquatus* [34], Tonkean macaques [30], and olive baboons [35]), which suggests different neuronal control for communicative and non-communicative gestures. These neuroanatomical and behavioral similarities between language lateralization and gesture production in several primate species, together with their phylogenetical proximity with *Homo sapiens* [36,37,38], led to the hypothesis that the brain lateralization for language control is inherited from a left-hemispheric specialization for intentional gestures in the common ancestor of humans and other catarrhine primates [39,40,41,42,43,44,45]. Thus, studying gestural laterality in primates seems particularly relevant to elucidate the proximate and ultimate causes of language lateralization.

Different hypotheses have been formulated concerning the emergence of manual laterality. Notably, the “postural origin theory” [46,47] proposes that manual laterality results from adaptations of the right hand for complex tasks in terrestrial species, free from postural constraints implied by arboreal locomotion. Some authors also hypothesized that manual laterality evidenced in captive non-human primates was the by-product of experimental biases and captivity (“artefactual hypothesis”) [48,49,50]. Looking specifically at communicative gestures, different factors might influence manual laterality in primates. Firstly, gestural laterality may depend on factors related to the interactional context, such as its emotional valence. Prieur and colleagues [26,29,51] evidenced that chimpanzees and gorillas preferentially use their right hands to gesture in aggression contexts, which may be explained by the hemispheric lateralization for emotions in vertebrates [52,53]. The relative position of the signaler and receiver during the interaction can also affect gestural laterality, as shown in chimpanzees and gorillas [26,29,54], presumably because of gesture directionality (which may imply a preferential use of the ipsilateral hand), and to asymmetrical emotional signals from recipient which could drive gesture production [26,55]. Secondly, gesture characteristics may affect hand preference, such as the type of gesture, as shown in chimpanzees and gorillas [25,26,29,54,56,57], or the sensory modality on which it is delivered (i.e., visual only, acoustic or tactile [26,54]). Moreover, in line with the “postural origin theory”, it can be hypothesized that the hand used to gesture depends on whether hands are engaged for signaler posture stability. This postural effect on laterality has notably been evidenced for manual tasks in red-capped mangabeys [58] and grey-cheeked mangabeys (*Lophocebus albigena*) [59]. Thirdly, sociodemographic characteristics of the signaler and receiver may affect gestural laterality. Although no or slight effects of dominance and kinship have been found in chimpanzees and gorillas [26,29,60], the age of the signaler affects gestural laterality in several species, as right-handedness increases with age (e.g., in chimpanzees [25,26,56]; gorillas [29,51]; olive baboons [32]).

To understand how all these factors may affect gestural laterality, it seems judicious to observe non-human primate gestures occurring in intraspecific contexts. Prieur and colleagues [26,29] were the first to adopt a multifactorial approach to characterize intraspecific gestural laterality, in chimpanzees and gorillas. To our knowledge, such method has never been adopted to study catarrhine monkeys’ gestural communication. Yet, right-handedness for communicative gestures have been evidenced in cercopithecid species (Tonkean macaques [30,31]; Campbell’s mona monkeys and red-capped mangabeys [34]), including for intraspecific gestures (olive baboons [32,33]), suggesting that it could be investigated beyond the great ape clade. Moreover, our understanding of the causes of gestural laterality, and, thus, to a greater extent our understanding of language evolutionary origins, benefits from comparisons between primates with different social systems and ecologies [61].

We present here a preliminary investigation of intraspecific gestural laterality in captive red-capped mangabeys, a semi-terrestrial monkey [62] originating from West African rainforest coastal regions [63,64,65,66]. They naturally live among multi-male, multi-female social groups, from 10 to 25 individuals [63,64], and exhibit linear hierarchy in captivity [67], with aggression and affiliation patterns both observed in despotic and relaxed macaque societies [68]. Recent results evidenced that captive red-capped mangabeys produce intentional body signals as part of their intraspecific communication, and, notably, manual gestures [69]. Experimental assessment of manual laterality in human-directed communication brought to light lateralized individuals for pointing gestures, and a dissociation of hand preference for communicative compared to non-communicative manual acts [34]. However, mangabeys’ intraspecific gestural laterality has never been studied. Our first aim here was to test which proximate factors would affect hand preference in red-capped mangabey gestures, by taking into account similar factors as those tested in multifactorial analyses in chimpanzees and gorillas [26,29], for comparison purposes. We tested the effects of the gesture production characteristics (sensory modality involved, signaler posture), of the interactional context (emotional valence of context, relative positions of signalers and receivers), and socio-demographic characteristics (age and sex of the signaler, relationship between signaler and receiver in terms of difference of age and sex, dominance, and kinship). Secondly, we tested whether captive red-capped mangabeys are lateralized at the individual and population-level for their intraspecific gestures. Based on previous findings in catarrhine apes and monkeys [25,26,28,32], we expected to find an overall right-hand bias in mangabey manual gesturing.

## 2. Materials and Methods

### 2.1. Subjects and Housing Conditions

We observed twenty-five captive red-capped mangabeys housed at the Station Biologique de Paimpont (University of Rennes 1, France). Subjects were 10 females and 15 males, including 3 juveniles, 14 middle-aged, and 8 old adults (Table 1). They lived in social groups, from two to 13 individuals, which have been stable from at least 8 months at the moment of data collection. Mangabeys were housed in outdoor-indoor enclosures of different sizes (from 8 to 26.4 m^2^ for indoor enclosures, 14.7 to 37.2 m^2^ for outdoor enclosures, and height from 2.5 m to 4.4 m) and could move in and out freely, using connecting tunnels. The temperature of indoor enclosures was set at 22 °C. Enclosures were enriched with perches made of wood and metal, on which swinging chains and hessian ribbons were attached. The floor of the indoor enclosures was covered with straw and sawdust, while the floor of the outdoor enclosures was covered with cement or bark. Feeding occurred twice a day, with fresh fruits and vegetables in the morning and monkey chows in the afternoon. Water was available ad libitum. 

### 2.2. Data Collection

#### 2.2.1. Focal Observations

We analyzed observational data collected from the end of January to the end of June 2018 for the purpose of describing red-capped mangabeys’ intraspecific signaling (see Reference [70]). We recorded focal videos for each monkey (“individual focal sampling method” [71]) using a JVC Full HD GZ-RX615 camcorder (JVCKENWOOD Europe B.V.). An observation session corresponded to 15 min of recording (mean session duration ± S.E. = 15.67 ± 0.10 min), and each individual was observed during 8 sessions. We, thus, totalized more than 2 h (125.33 ± 0.75 min) of recording per individual, and 52.22 h of observations overall. Sessions were scheduled randomly, and then equilibrated so that all monkeys were observed at all times of day (from 9:00 a.m. to 6:00 p.m.) and at all feeding periods (before or during the first feeding/between two feedings/after or during the second feeding). BORIS v.6.0.6 software [72] was used to point each event of manual gesture and each social interaction involving the focal individual. We then coded parameters and behaviors associated to each gesture occurrence (i.e., the signaler and receiver identities, the type and characteristics of gesture, the social context, and relative position of interactants).

#### 2.2.2. Brachio-Manual Gestures

We recorded every instance of unimanual gestures produced by the focal individuals, in intraspecific dyadic context. A brachio-manual gesture was defined as any movement of one arm or hand that was: (i) nonlocomotor and “mechanically ineffective”, though not excluding gestures that implied contact with a substrate or the recipient [15,73,74]; (ii) physically directed to a receiver, as shown by the signaler’s head or body orientation and/or physical contact between the signaler and the receiver [12,15,54,56,69,70,75,76], and/or preceded by “audience checking” within the 5 s before the gesture [17,70]; (iii) adapted to the receiver attention, i.e., produced in front of a visually attentive recipient (the signaler being more or less 45° in front of the receiver’s face), except for tactile and audible gestures [15,69,70,76,77,78]; and (iv) followed by a voluntary response of the receiver within 5 s [70,76,79,80].

#### 2.2.3. Emotional Valence of Context

The social contexts of gesture production were determined based on the signaler and receiver’s behavior within the 5 s before and after the signal [70,81]. Six different contexts were distinguished, and then categorized according to their assumed emotional valence (Table 2). Aggression and submission contexts were assumed to be “negative”, in contrast to affiliation, grooming, social play and socio-sexual contexts that were qualified as “positive or neutral”. We note that the latter contexts may involve ambiguous behaviors, falling within “positive” contexts, while possibly eliciting negative emotions in interactants.

#### 2.2.4. Relative Positions of Interactants

For each gesture instance, we also recorded the relative positions of the signaler and the receiver in each other’s visual field [26,29,51,54,82]. The receiver position was coded as “front” if it was positioned more or less 60° in front of either signaler’s face [83], and “right” or “left” depending on whether it was in the signaler’s right or left visual field. The position of the signaler was similarly coded from the receiver’s point of view.

#### 2.2.5. Gesture Characteristics and Signaler’s Posture

For each gesture occurrence, we noted the hand used (left or right), the type of gesture produced [69,70], and the sensory modality on which it could be perceived. Gestures were classified as “visual” if they were silent and distant brachio-manual movements, “audible” if they provoked a sound, and “tactile” if they involved a physical contact with the receiver [16,69,70,77,84,85]. According to the “postural” theory on gestural laterality origins [46,47], signaler posture may constrain the availability of hands to gesture and, thus, be a determining factor shaping gestural laterality. To test for this effect in red-capped mangabeys, we categorized the signaler’s posture when gesturing, depending on whether one hand was “engaged” in posture as a support (subject walking or standing quadrupedally, standing bipedally with hand on substrate, climbing or hanging on cage) or both hands were “free” (subject sitting, laying, jumping, or standing bipedally without other support).

#### 2.2.6. Signaler and Receiver Relationships

In order to assess the effect of social factors on the hand used to gesture, we characterized the relationship between the signaler and the receiver in terms of dominance and kinship. Dominance was deduced from avoidance behaviors [86], i.e., turning away from another individual, avoiding an individual by changing direction, avoiding contact by moving a body part away from the proximity of another individual, or fleeing an individual by walking or running in opposed direction [87]. All avoidance behaviors involving focal subjects were analyzed, and, when at least 3 occurrences were observed for a dyad, we computed the percentage of avoidance of subject A from subject B over the total number of these behaviors between A and B [88]. Subject A was considered subordinate to subject B if this percentage was above 50%. Moreover, kinship was known for all individuals in the colony, and the signaler and the receiver were considered as “kin” if they were first-degree relatives (parents or siblings). Finally, we noted whether the signaler and the receiver were of same age or not (based on age categories: juveniles, middle aged, and old adults) and of same sex or not. 

### 2.3. Statistical Analyses

We used R v.3.6.2 [89] for all statistical analyses. All tests were two-tailed, and alpha-level was set at 0.05. 

Firstly, we tested which factors affected hand use in red-capped mangabey gestural communication, among contextual factors (valence of social context, signaler and receiver positions in each other’s visual field), gesture production characteristics (gesture sensory modality, signaler posture), and socio-demographic factors (dominance and kinship between the signaler and receiver, signaler age category and sex). To do so, we used a Generalized Linear Mixed Model (GLMM) of the binomial family to analyze the hand used to produce gestures (right or left), depending on the above-cited variables, and including the identity of the signaler as a random effect (R package {lme4}). The model quality was verified by checking for the absence of data overdispersion, the independence of model residuals, and the absence of fixed factors collinearity (R packages {RVAideMemoire} and {performance}). 

Secondly, we tested the presence of an overall gestural laterality in our captive red-capped mangabeys. To assess gestural laterality at the individual level, for each individual that produced at least 6 manual gestures, we performed binomial tests to compare the proportion of right- and left-hand gestures to a theoretical proportion of 0.5. For each of these subjects, a handedness index (HI) was also computed using the following formula: HI = (R − L)/(R + L), where R corresponds to the number of gestures produced with the right hand and L with the left hand. This index, between −1 and 1, is commonly used to assess laterality [90], as its sign reveals the potential bias direction: toward the right if it is positive, and the left if it is negative. We tested whether HI differed from null values using a Wilcoxon signed-rank test. The strength of individuals’ hand preference was estimated by the absolute value of HI (ABSHI). Gestural laterality was assessed at the population level based on the proportion of lateralized individuals in the population and the average ABSHI value. Moreover, we compared the number of gestures that mangabeys produced with the right or left hand using a Wilcoxon signed rank test.

## 3. Results

### 3.1. Manual Gestures

We recorded 275 occurrences of unimanual gestures produced in intraspecific dyadic contexts by captive red-capped mangabeys. Twenty of the 25 captive mangabeys produced manual gestures, and 10 different gestures were observed (Table 3), of which 7 have been previously described as intentional gestures [69].

### 3.2. Factors Affecting Hand Use in Gestural Communication

**Figure 1:**
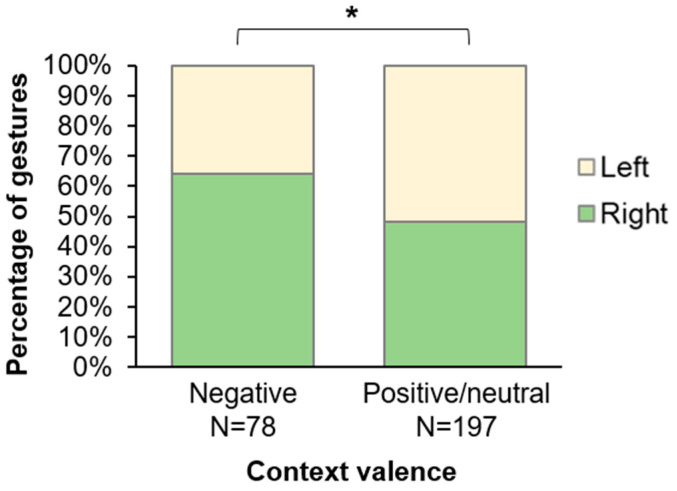
Percentage of gestures produced with the left or right hand, depending on the emotional valence of context. “Negative” valence corresponds to aggression and submission contexts, and “positive/neutral” corresponds to affiliation, grooming, play, and sexual contexts. GLMM Binomial: *: *p* < 0.05.

**Figure 2:**
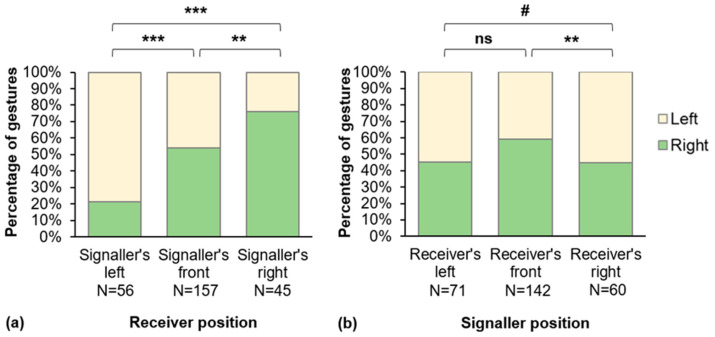
(**a**) Percentage of gestures produced with the left or right hand, depending on the receiver position in the signaler’s visual field; (**b**) Percentage of gestures produced with the left or right hand, depending on the signaler position in the receiver’s visual field. GLMM Binomial: ns: non-significant difference; #: 0.05 < *p* < 0.1; **: *p* < 0.01; ***: *p* < 0.001.

Contextual factors affected the hand they used to communicate (GLMM Binomial, detailed comparison results in Table 4). Particularly, we found a significant effect of the emotional valence of context in which the gestures were produced (GLMM Binomial, Type II Wald Chi-square test: *X*^2^_1_ = 6.383, *p* = 0.011) with more right-hand gestures in “negative” contexts than in others (Figure 1). Moreover, the relative receiver and signaler’s positions had a significant effect on the hand used to gesture (receiver position: *X*^2^_2_ = 29.233, *p* < 0.001; signaler position: *X*^2^_2_ = 7.068, *p* = 0.029). Signalers adapted the side used to communicate to the position of the receivers, using more the right hand when the receivers were in their right visual field and conversely (Figure 2a). Moreover, they used less the right hand when they gestured in the right visual field of receivers than when they were in front of them (Figure 2b). Interestingly, we found no effect of the sensory modality of gestures on hand use (*X*^2^_2_ = 3.148, *p* = 0.207), nor of the signaler posture (*X*^2^_1_ = 1.373, *p* = 0.241) or socio-demographic factors (Dominance: *X*^2^_2_ = 0.500, *p* = 0.779; Kinship: *X*^2^_1_ = 0.005, *p* = 0.941; Age difference: *X*^2^_1_ = 1.670, *p* = 0.196; Sex difference: *X*^2^_1_ = 0.065, *p* = 0.799; Signaler’s age: *X*^2^_2_ = 0.718, *p* = 0.698; Signaler’s sex: *X*^2^_1_ = 0.246, *p* = 0.620).

### 3.3. Gestural Laterality at the Individual and Population Level

None of the red-capped mangabeys were significantly lateralized for their manual gestures (Binomial test, Table 5). Thus, there was no laterality bias at the population-level. The HI did not overall differ from null values (Wilcoxon signed rank test: *N* = 11, *V* = 30, *p* = 0.823), and the mean ABSHI value was low (ABSHI ± S.E. = 0.24 ± 0.04), as mangabeys produced as much manual gestures with the right than with the left hand (Wilcoxon signed rank test: *N* = 20, *V* = 98, *p* = 0.320).

## 4. Discussion

We present here a preliminary assessment of captive red-capped mangabey intraspecific gestural laterality. We found a significant effect of interactional context factors on the hand used by red-capped mangabeys to communicate, corroborating previous findings from ape studies and suggesting an effect of emotional lateralization on gestural communication. On the contrary, factors related to gesture production and socio-demographic characteristics of the signaler and the receiver did not affect mangabey gestural laterality. None of the individuals we observed were significantly lateralized when looking at their overall gesture production; thus, no population-level bias was observed, which we discuss with regards of our sample size.

We found that contextual factors affected the hand used by red-capped mangabeys to communicate with their conspecifics. As found in chimpanzees and gorillas [26,29,51], mangabeys used their right hand more to gesture in contexts associated with negative emotional states (i.e., aggression and submission contexts). At first sight, these findings may seem to contradict theories on brain asymmetries for emotion processing, which would predict a left-hand preference for negative contexts [52]. The “right hemisphere theory” indeed proposes that the right hemisphere controls perception and expressions of emotions [91,92,93], and the “valence theory” suggests that the right and left hemispheres, respectively, control negative emotions, frequently associated with withdrawal behaviors, and positive emotions, frequently associated with approaches [94,95,96]. Prieur and colleagues hypothesized that the observed right-hand bias of apes in negative contexts originates from the same mechanisms that underlie left prefrontal brain region activation in humans during negative events, such as aggressions [97]. Taking the perspective that aggression contexts involve not only negative emotions (e.g., anger) but also motivation for “approaching” behaviors [97], this seems actually in accordance with the “approach/withdrawal” theory on brain asymmetries [52,97,98,99], which could explain results on chimpanzees, gorillas, and red-capped mangabey gestures. Aside from the effect of the context valence, we found that gestural laterality was significantly affected by the relative positions of the signaler and the receiver. Red-capped mangabeys preferentially used their ipsilateral hand to gesture toward conspecifics, probably to more efficiently direct their signal. This is in accordance with observation of captive chimpanzees and gorillas [26,29,54], who preferentially performed visual and tactile gestures with their right hand when their recipient was in their right visual field. These results highlight the importance of taking into account the receivers’ positions when studying intraspecific gestural laterality of primates, or to control this parameter in experimental conditions, given its substantial effect on hand use.

Factors related to the gesture production in itself did not affect gestural laterality, since we found no effect of the sensory modality involved (visual only, acoustic or tactile) nor of the signaler posture while gesturing. Experimental studies brought to light an effect of red-capped mangabeys’ postures for manual tasks, and this was particularly true for complex tasks [58]. We can hypothesize that this postural effect is not found for communicative gestures because they are less demanding in terms of movement precision, compared for instance to bimanual manipulation tasks (“task complexity hypothesis” [100]). Interestingly, socio-demographic characteristics of the signaler and the receiver had no effect on gestural laterality. The signaler’s sex did not affect the hand used to gesture, as found in apes and olive baboons [28,32,33,35,60]. Although right-hand preference has been found to increase with age in apes and olive baboons [25,26,29,32,51,56], we did not find this effect in red-capped mangabeys. This is not surprising regarding the absence of lateralized individuals in our population, but these aspects should be further investigated with an increased sample size. Finally, the absence of dominance or kinship effect on mangabey gestural laterality was in accordance with the results obtained from ape studies [29,60]. One study showed an effect of social hierarchy on gestural laterality of primates, describing more right-handedness in subordinate captive chimpanzees than in other individuals [26], and this was hypothesized to result from the higher level of psychological stress in these individuals, in line with the effect of negative context on right-handedness. We only analyzed here the effect of dominance relationships determined at the dyadic level, but future studies with larger samples within a same social group may permit to test the effect of mangabeys’ individual hierarchical ranks on gestural laterality. A previous study evidenced an effect of social hierarchy on social laterality in captive mangabeys: the individuals that were approached more frequently from their left side were the ones with higher social ranks [83]. As we show here that monkeys’ relative positions affect the hand used to gesture, we may hypothesize an indirect effect of mangabeys’ social ranks on gestural laterality. Social status has been shown to be related to behavioral lateralization in diverse species (e.g., in humans [101,102] but also in more evolutionary distant species, such as geckos, *Ptyodactylus guttatus* [103]), which may reflect some social benefits conferred by lateralization at the individual level.

Looking at the production of brachio-manual gesture as a whole, we found no significant gestural lateralization at the individual nor population level in captive red-capped mangabeys. Given that population-level right handedness for communicative gesture was found in several catarrhine monkey species [30,31,32,33], we hypothesize that the absence of gestural lateralization found here results from our sample size, which is the main limit on research on behavioral laterality [104]. On one hand, the small number of data points per subjects could hide existing individual biases [27]. On the other hand, the number of subjects may be not sufficient to reveal a population-level bias [105,106]. A previous study evidenced right-handed mangabeys for human-directed pointing gestures, yet this was found only for the individuals with high referential communication abilities, suggesting substantial inter-individual variability for this trait [34]. Present results should be completed by measures on an increased sample, to confirm or contradict this hypothesis. This should also permit to investigate the effect of subjects’ characteristics (such as age, sex, or hierarchical rank) on gestural lateralization at the individual level. Moreover, it should permit to analyze independently the different types of gestures produced by mangabeys, as gesture type could affect the direction and strength of laterality [25,26,29,54,56,57].

## 5. Conclusions

This first investigation of captive red-capped mangabey gestural laterality corroborates previous findings from ape gesture studies, particularly regarding the effect of interactional context on hand use, which suggests that emotional lateralization affects manual preference in non-human primate gestural communication. We did not highlight any bias at the individual or population level, possibly because of our sample size. Thus, these promising results should be completed with further observational studies of mangabey gestures, and by similar investigations in other monkey genera, with the view to understand the evolutionary roots of modern human language and handedness.

## Figures and Tables

**Table 1:** Characteristics of the red-capped mangabeys.

Social Group	Individual’s Name	Sex	Date of Birth	Age Category
I.	Bell	Female	31 March 2002	Old adult
	Chipie	Female	28 June 1992	Old adult
	Gofrette	Female	8 November 1996	Old adult
	Joly	Female	22 October 2000	Old adult
	Zunie	Female	3 July 1987	Old adult
	Chipse	Female	3 January 2006	Middle-aged
	Julie	Female	8 May 2004	Middle-aged
	Maillette	Female	29 December 2009	Middle-aged
	Many	Female	14 August 2008	Middle-aged
	Triskelle	Female	21 April 2015	Juvenile
	Kargi	Male	19 May 2005	Middle-aged
	Litchi	Male	20 April 2015	Juvenile
	Pouët	Male	14 March 2015	Juvenile
II.	Kamel	Male	7 September 2010	Middle-aged
	Roby	Male	18 November 2010	Middle-aged
III.	Bandit	Male	15 June 1991	Old adult
	Coët	Male	31 August 2011	Middle-aged
	Tips	Male	10 July 2011	Middle-aged
IV.	Pirate	Male	17 October 1992	Old adult
	Carillon	Male	2 April 2007	Middle-aged
	Elky	Male	6 November 2009	Middle-aged
	George	Male	5 June 2006	Middle-aged
V.	Marti	Male	16 October 1998	Old adult
	Isba	Male	20 April 2004	Middle-aged
	Lenni	Male	7 October 2006	Middle-aged

**Table 2:** Social contexts of gestures (adapted from Reference [70]).

Valence	Context	Associated Behaviors
Positive or neutral	Affiliation	Physical proximity between subjects (at less than one arm length), calm approach of recipient or gentle physical contact.
	Grooming	Grooming, tactile examination
	Social play	Play-fight, rough or gentle (involving manual fighting, biting, gentle or rough touching and grabbing), or locomotor-rotational play (play with few physical contacts, but involving pursuits, jumps, somersaults)
	Socio-sexual	Touching, smelling, licking genital part, or mounting
Negative	Aggression	Physical aggression of the recipient by the signaler (biting, beating, rough manipulation), flight or avoidance of the signaler by the recipient, or intergroup conflict in which the signaler and recipient were in different social groups
	Submission	Flight or avoidance of recipient by signaler

**Table 3:** Manual gestures observed in captive red-capped mangabeys (adapted from Reference [70]). *Nb*: number of instances; *N*: number of different signalers; *: previously described as intentional gestures [69].

Gesture	Description	Sensory Modality	*Nb*	*N*
Embrace *	Signaler puts one arm around the receiver’s body.	Tactile	13	7
Grab body part *	Signaler closes one hand on a receiver’s body part.	Tactile	144	17
Grabbing movement *	Signaler throws one arm in the receiver’s direction, with hand closing at the end of the movement.	Visual	9	4
Pull body part	Signaler holds and pulls a receiver’s body part.	Tactile	6	3
Push	Signaler pushes the receiver away.	Tactile	1	1
Slap object *	Signaler slaps cage element or ground with an open hand.	Audible	16	6
Slap receiver *	Signaler hits a receiver’s body part with an open hand.	Tactile	14	6
Slap self	Signaler hits itself with an open hand, in a unique or repeated movement.	Visual	2	1
Touch *	Signaler gently puts one open hand on receiver’s body.	Tactile	1	1
Throw arm *	Signaler throws one arm in the receiver’s direction.	Visual	69	18

**Table 4:** Fixed effects of the GLMM Binomial. The first factor cited for each comparison corresponds to the reference factor. Positive values of the estimate indicate that the reference factor drives higher proportion of right-hand gestures, and conversely. *: *p* < 0.05; **: *p* < 0.01; ***: *p* < 0.001.

Fixed Effects	Comparison	Estimate	Standard Error	*z*	*p*
Context valence	Negative vs. Positive/neutral	1.111	0.440	2.526	0.012 *
Receiver position	Signaler’s right vs. left	2.945	0.549	5.363	<0.001 ***
	Signaler’s right vs. front	1.576	0.435	3.626	<0.001 ***
	Signaler’s left vs. front	−1.369	0.467	−2.931	0.003 **
Signaler position	Receiver’s right vs. left	−0.846	0.472	−1.792	0.073
	Receiver’s right vs. front	−1.117	0.423	−2.643	0.008 **
	Receiver’s left vs. front	−0.272	0.407	−0.667	0.505
Sensory modality	Audible vs. Tactile	0.150	0.690	0.217	0.828
	Audible vs. Visual	−0.523	0.693	−0.754	0.451
	Tactile vs. Visual	−0.673	0.383	−1.754	0.079
Posture	“Engaged” hand vs. “free”	−0.332	0.440	−1.172	0.241
Dominance of signaler	Dominant vs. subordinate	0.112	0.328	0.342	0.732
	Dominant vs. unclear	−0.303	0.582	−0.521	0.603
	Subordinate vs. unclear	−0.415	0.596	−0.696	0.486
Kinship	Non-kin vs. kin	0.029	0.395	0.073	0.941
Age difference	Different vs. same	−0.681	0.527	−1.292	0.196
Sex difference	Different vs. same	0.111	0.434	0.255	0.798
Signaler age category	Juvenile vs. middle-aged	−0.153	0.366	−0.418	0.676
	Juvenile vs. old adult	−0.580	0.685	−0.846	0.397
	Middle-aged vs. old adult	−0.427	0.613	−0.696	0.486
Signaler sex	Female vs. male	0.236	0.476	0.496	0.620

**Table 5:** Nb: number of unimodal gestures; Prop.R: proportion of gestures produced with the right hand; HI: handedness index; *p*: binomial test *p*-values.

Signaller	Sex	Age Category	Nb	Prop.R.	HI	*p*
Bell	Female	Old adult	7	0.71	0.43	0.453
Chipie	Female	Old adult	2	-	-	-
Gofrette	Female	Old adult	7	0.71	0.43	0.453
Joly	Female	Old adult	5	1.00	-	-
Zunie	Female	Old adult	13	0.69	0.38	0.267
Chipse	Female	Middle-aged	4	0.25	-	-
Julie	Female	Middle-aged	11	0.45	−0.09	1
Maillette	Female	Middle-aged	1	-	-	-
Many	Female	Middle-aged	12	0.33	−0.33	0.388
Triskelle	Female	Juvenile	8	0.63	0.25	0.727
Pirate	Male	Old adult	4	0.50	-	-
Coët	Male	Middle-aged	17	0.59	0.18	0.629
Isba	Male	Middle-aged	5	0.60	-	-
Kamel	Male	Middle-aged	2	-	-	-
Kargi	Male	Middle-aged	21	0.57	0.14	0.664
Lenni	Male	Middle-aged	4	1.00	-	-
Roby	Male	Middle-aged	19	0.63	0.26	0.359
Tips	Male	Middle-aged	2	-	-	-
Litchi	Male	Juvenile	61	0.44	−0.11	0.443
Pouet	Male	Juvenile	70	0.47	−0.06	0.720

## Data Availability

The data presented in this study are openly available as a supplementary table (Supplementary Table S1).

## Supplementary Materials

The following are available online at https://www.mdpi.com/article/10.3390/ani12020186/s1, Table S1: Analyzed data on red-capped mangabeys’ gestural laterality.
